# Valorization of Expanded Polystyrene by Embedding of High GFRP Loading Through Cold-Mixing Solvent-Assisted Process

**DOI:** 10.3390/polym18131567

**Published:** 2026-06-24

**Authors:** Federico Olivieri, Stefano Scognamiglio, Roberto Avolio, Rachele Castaldo, Mariacristina Cocca, Gennaro Gentile, Silvia Olivotto, Maria Emanuela Errico

**Affiliations:** 1 Institute for Polymers, Composites and Biomaterials, National Research Council of Italy, Via Campi Flegrei 34, 80078 Pozzuoli, Italy; federico.olivieri@cnr.it (F.O.); stefano.scognamiglio@stems.cnr.it (S.S.); rachele.castaldo@cnr.it (R.C.); mariacristina.cocca@cnr.it (M.C.); gennaro.gentile@cnr.it (G.G.); mariaemanuela.errico@cnr.it (M.E.E.); 2 Enel Green Power S.p.A., Viale Regina Margherita 125, 00198 Rome, Italy; silvia.olivotto@enel.com

**Keywords:** solvent-assisted processing, GFRP recycling, high filler loaded composites

## Abstract

The increasing accumulation of glass-fiber-reinforced polymer (GFRP) waste poses significant environmental challenges, calling for effective and scalable recycling strategies. In this work, a solvent-assisted cold mixing process was employed to incorporate very high amounts of GFRP (up to 75 wt%) into recycled expanded polystyrene (ePS). The composites were deeply characterized, with particular attention to the role of particle size distribution and filler content. The results demonstrated that GFRP granulometry played a key role in determining composite performance. Intermediate particle sizes (0.25 mm) provided the best balance between dispersion, interfacial interaction, and mechanical properties, whereas excessively fine fractions introduced defects and reduced impact resistance (from 0.7 to 2.0 kJ/m^2^ going from dust to 0.25 mm at 75 wt%). Notably, the solvent-assisted approach has been widely recognized as an effective strategy to ensure homogeneous dispersion even at high filler contents, allowing subsequent melt processing without re-agglomeration. Recycled composites retained most of their chemical and mechanical properties after reprocessing, with only moderate performance losses mainly related to fiber fragmentation. Overall, this study demonstrates an effective and sustainable route for the simultaneous valorization of ePS and GFRP waste, enabling the production of highly loaded composites with preserved functionality and improved resource efficiency.

## 1. Introduction

Wind energy represents one of the most promising renewable and clean energy sources for reducing greenhouse gas emissions, which largely originate from the consumption of fossil fuels [1]. Although wind turbines are already about 80–85% recyclable [2], mainly due to the recovery of steel and other metals, the management of end-of-life (EoL) wind turbine blades (WTBs) still poses key challenges [2,3]:

- Achieving full circularity across all turbine components;
- Moving beyond landfilling and energy recovery toward high-value recycling pathways;
- Establishing a robust and economically viable business case for recyclers and off-takers, which is currently lacking and limits the scalability of the recycling value chain.

WTBs are typically composed of three main sections (root, midspan, and tip), each characterized by distinct structural configurations. The root section generally consists of a cylindrical structure reinforced with glass-fiber-reinforced resin, while the midspan and tip feature a lightweight “sandwich” construction. This structure is composed of two thin composite panels enclosing a lightweight core material (commonly balsa wood or polyvinyl chloride), bonded together with an adhesive [4]. Overall, WTBs are primarily made of polymer matrix composites (such as epoxy or polyester resins) reinforced with glass fibers (GFs), carbon fibers (CFs), or their hybrids [5]. These materials provide high fatigue resistance and mechanical strength due to their cross-linked matrix structures. Typically, WTBs contain about 60–75 wt% reinforcing fibers, 20–35 wt% polymer matrix, 2–10 wt% foam core materials and adhesive, and 1–5 wt% metals [6].

The cross-linked nature of the resin and strong adhesion with the fibers make composite materials difficult to separate and recycle. Furthermore, the relatively low value of recovered glass fiber reduces the economic incentive for recycling [7]. As a result, most EoL WTBs have historically been disposed of in landfills or incinerated, leading to resource loss and additional environmental impacts [8].

In recent years, various alternative EoL management strategies for WTBs have been explored within the broader framework of the circular economy [9]. These strategies align with the principles of narrowing, slowing, and closing resource loops, aiming to minimize resource use, extend product lifespans, and recover valuable materials. Current research focuses on three main approaches: repurposing, recycling, and recovery [10]. Repurposing involves reusing blades or blade components in new applications (e.g., noise barriers, pedestrian bridges, or urban furniture), preserving material value and reducing processing requirements. However, due to their large size, complex geometry, and solid structure, large-scale repurposing remains challenging [11].

When direct reuse is not feasible, recycling and recovery become essential pathways for WTB waste management [12]. Recycling methods for fiber-reinforced polymer composites can be broadly divided into mechanical, thermal, and chemical processes. Mechanical recycling, a relatively mature and low-cost technology, involves size reduction through shredding, crushing, or milling to produce fibrous or powdered materials for reuse, often in construction composites [13]. However, conventional mechanical recycling routes are highly energy-intensive and typically lead to significant fiber shortening and limited control over fiber–matrix interactions, which ultimately restricts the performance of the recycled material [14]. Thermal recycling, such as pyrolysis, decomposes the polymer matrix to recover fibers and fuels, though high energy consumption, complex product streams, and pollutant emissions limit its economic viability. Chemical recycling, involving depolymerization in sub- or supercritical fluids, can recover fibers and monomeric components but suffers from high energy demands and complex degradation products [15,16,17,18,19].

Given the large size, high strength, and heterogeneous composition of WTBs, pretreatment through mechanical processes is often required before recycling or recovery. Cutting techniques, such as diamond wire or circular saws, can segment blades into smaller, manageable pieces, although these processes are energy-intensive and generate dust and noise emissions [20]. Further crushing can produce composite particles suitable for use in construction materials, particularly in concrete applications, enabling large-scale utilization of WTB waste. However, challenges remain, including inefficient processing of non-composite components (e.g., PVC, balsa wood, polyurethane) [21,22,23].

To achieve more sustainable management of EoL WTBs, it is crucial to develop intelligent, low-emission cutting and crushing technologies, along with innovative material reuse strategies that maximize resource recovery while minimizing environmental impacts [24]. Advancing these technologies will play a key role in steering the wind energy sector toward circularity and long-term sustainability [25].

In recent years, a novel technique has been developed and refined for the production of highly filled thermoplastic composites through solvent-assisted fluidization of various polymer matrices [26], including recycled polymers. This cold-mixing process is both energy-efficient and effective. Its performance has been successfully demonstrated using recycled expanded polystyrene (ePS) [27], widely available as recycled polymeric material due to its use in numerous high-volume applications, which can retain good mechanical properties even after multiple recycling cycles [28,29]. This approach represents a significant departure from conventional mechanical recycling routes, as it enables the incorporation and dispersion of recycled composite fractions without the need for extensive additional size reduction, thereby limiting further fiber damage and reducing overall energy consumption [25,26]. Recent studies have also highlighted that the intrinsic difficulty in separating fiber and matrix phases in thermoset-based composites remains a key limitation for conventional recycling approaches, further motivating alternative low-energy strategies [30]. In this work, we focused on the development and optimization of mechanical recycling processes for glass-fiber-reinforced thermoset composites recovered from decommissioned EoL WTBs. The study was structured in an initial phase aimed at defining the optimal grinding and sieving conditions for the glass-fiber-reinforced polymer (GFRP) composites and at characterizing the resulting fractions. Based on these results, a solvent-assisted cold mixing process was designed and optimized, combining the recycled GFRP fractions at varying proportions. Thermoplastic PS composites incorporating GFRP were produced with a high filler content of up to 75 wt%. This method enables the use of recycled ePS as the polymeric phase, thereby enhancing the environmental sustainability of the production process. The resulting materials were characterized through mechanical, thermal, spectroscopic, and morphological analyses. Finally, the recyclability of the most promising material was further demonstrated, confirming the potential for the implementation of circular approaches to the management of polymeric materials at their end of life.

## 2. Materials and Methods

GFRP panels were supplied by Enel Green Power SpA (Rome, Italy) in the form of large chunks cut from dismantled WTBs. ePS from post-consumption was obtained from local waste treatment plants. Acetone (technical grade) was supplied by Merck (Darmstadt, Germany).

### 2.1. GFRP Pre-Treatment

The GFRP panels were cut into sections of approximately 2–4 × 2–4 cm with a diamond blade to allow efficient loading into the mill.

The GFRP fragments were then ground using a Retsch SM100 cutting mill (Retsch, Haan, Germany) equipped with a 129.5 mm rotor with 3 steel cutting blades operating at a fixed rotation rate of 1500 rpm. Bottom screens with nominal aperture sizes of 1 mm and 0.25 mm were used. GFRP was charged into the mill in small batches (approximately 30–40 g, corresponding to 2–3 GFRP fragments), which were allowed to undergo complete fragmentation and discharging through the screen before inserting a new batch. The approximate residence time of the material in the milling chamber was <1 min with the 1 mm screen and approximately 3 min with the 0.25 mm screen. During both cutting and milling, the GFRP dust produced was collected using a dedicated suction system for subsequent reuse. The cutting powder accounted for a relevant amount, approximately 17 wt%, of the starting material; it was then recovered and used for subsequent characterizations and composite production, labeled as “dust”.

The particle size distribution of the ground materials was defined by sieving using metallic meshes with apertures ranging from 1 mm to 100 µm and a Retsch AS 200 vibratory sieve shaker (Retsch, Haan, Germany). For the sieving, batches of 200 g of each sample (GFRP ground with 1 mm bottom sieve, GFRP ground with 0.25 mm bottom screen, dust) were placed onto the top sieve, and then the vibratory shaker was started and allowed to run for 1 h at a 60% amplitude setting (corresponding to approximately 1.8 mm). The total amount of material sieved was 600 g for the 1 mm and 0.25 mm fractions and 400 g for the dust fraction.

### 2.2. Solvent-Assisted Composite Preparation

PS was swollen in acetone to form a viscous gel-like material, using a weight/volume ratio of 1 g polymer per 1 mL acetone (1:0.79 wt/wt). Acetone and the filler were added gradually to promote the swelling of the polymer, which, in turn, facilitated an effective and homogeneous dispersion of the filler within the PS. In particular, 50 wt% or 75 wt% of GFRP, calculated based on the total dry weight of the polymer and filler, was added to the mixture. The mixtures were stirred for 15 min at room temperature and then left overnight to allow the acetone to evaporate. Subsequently, the compounds were treated in an oven at 40 °C under vacuum to remove any residual acetone, resulting in dry compounds. For comparison, a recycled PS sample was subjected to the same experimental procedure.

The specific codes and compositions of the compounds are detailed in Table 1. For comparison, a pure PS sample was also prepared by swelling it in acetone and subsequently drying it, following the same procedure used for composite fabrication.

The dried materials were molded using a Dr Collin P200E heating press (Ebersberg, Germany). The materials were heated at 200 °C and subjected to a pressure gradient. Initially, they were held at zero pressure for 5 min, then the pressure was raised to 50 bar for 2 min, followed by 100 bar for an additional 2 min, and finally, to 200 bar for 1 min. Then, the composites were quickly cooled to room temperature by circulating cold water through the press plates before releasing the pressure. The resulting sheets had lateral dimensions of 60 × 60 mm and a thickness of approximately 3 mm. Specimens were then prepared from these sheets for flexural and impact testing, as well as for thermal and morphological analyses.

It should be noted that no compatibilizer or surface treatment was employed in this study; therefore, the observed interfacial features reflect the intrinsic interaction between the recycled polystyrene matrix and the filler under solvent-assisted processing conditions.

### 2.3. Recycled Composite Preparation

The PS_75_0.25mm sample was selected to test its further recyclability by conventional melt mixing. To this aim, the material was processed in a Brabender Plastograph EC internal mixer (Brabender GmbH & Co. KG, Duisburg, Germany) at 200 °C, 60 rpm. The recycled material was coded as PS_Rec. After mixing, the material was pelletized and hot-pressed (same thermal conditions described above) to produce sheets approximately 3 mm thick, which were then used for flexural and impact resilience tests.

### 2.4. Characterization and Methods

The determination of the fiber/polystyrene ratio of the GFRP was performed by calcinating the organic part by heating 5 fragments of GFRP (for a total weight of approximately 100 g) up to 700 °C in an oxidizing atmosphere, gradually increasing the temperature in steps of 100 °C per hour.

The ground materials and the dust collected during cutting were analyzed by scanning electron microscopy (SEM) using an FEI Quanta 200 FEG (FEI, Eindhoven, The Netherlands), by means of a secondary electron detector and an accelerating voltage of 10 kV. Before the analysis, the samples were coated with a gold–palladium layer (about 10 nm thick) by means of an Emitech K575X sputter coater (Quorum Technologies, Lewes, UK). Energy-dispersive X-ray (EDX) analysis was performed using an Inca Energy System 250 and an Inca-X-act LN2-free analytical silicon drift detector (Oxford Instruments, Abingdon-on-Thames, UK) integrated into the SEM equipment. Moreover, the morphology of the prepared composites was analyzed using the above-described SEM equipment and procedure, observing cryogenically fractured cross-sections.

A quantitative estimation of particle shape was obtained by visually analyzing SEM images and approximating the characteristic length (L) and diameter (D) of fibers and particles. For elongated particles, the aspect ratio (L/D) was estimated, while for the dust fraction, consisting mainly of equiaxed particles, an equivalent aspect ratio was considered. Distributions were derived from multiple SEM observations, allowing the estimation of mean, median, and weighted average aspect ratios for each fraction. The weighted average was defined to qualitatively account for the higher contribution of longer fibers, which are more relevant for stress transfer.

GFRP fragments were analyzed by Fourier transform infrared (FTIR) spectroscopy in attenuated total reflectance (ATR) mode using a PerkinElmer Spectrum One FTIR spectrometer (Shelton, CT, USA) equipped with an ATR module, using a resolution of 4 cm^−1^ and 32 scan collections.

Solid-state ^13^C Nuclear Magnetic Resonance (NMR) was performed using a Bruker Avance II 400 spectrometer (Bruker Biospin Co., Billerica, MA, USA) operating at a static field of 9.4 T, equipped with a 4 mm magic angle spinning (MAS) probe. GFRP samples were packed in 4 mm zirconia rotors sealed with Kel-F caps and spun at 10 kHz. Spectra were recorded in cross-polarization, with a ^1^H π/2 pulse width of 3.9 μs, a ^1^H–^13^C contact time of 2 ms and a high-power proton decoupling pulse scheme during acquisition, averaging 20,000 scans.

Differential scanning calorimetry (DSC) analyses were performed on a TA-Q2000 instrument (TA Instrument, New Castle, DE, USA) to investigate the thermal behavior of PS and PS_75_0.25mm, representing the highest filler loading investigated in this work. Measurements were carried out under a nitrogen atmosphere to minimize oxidative degradation. Samples of approximately 5 mg were placed in non-hermetic aluminum pans to allow pressure equilibration during heating. All samples were subjected to a heating–cooling–heating cycle at a constant rate of 20 °C/min. The first heating scan was performed from room temperature to 250 °C to erase thermal history, followed by cooling to room temperature and a second heating scan under identical conditions. The glass transition temperature (T_g_) was determined from the second heating scan as the midpoint of the heat flow step transition. Three-point flexural tests were performed using an Instron 4505 machine (ITW group, Glenview, IL, USA) at a deformation speed of 2 mm/min, with a span length of 48 mm and specimen dimensions of 8 mm in width and 3 mm in thickness (cross-sectional area of 24 mm^2^), resulting in a span-to-depth of 16:1, to evaluate the flexural modulus and flexural strength according to the ASTM D790 test method [31]. For each sample, five specimens were tested, and the average and standard deviation values of flexural modulus, peak stress, and strain at break were collected. To provide a benchmark for reinforcement efficiency, the elastic modulus of the composite was estimated using the rule of mixtures and the Halpin–Tsai model. Calculations were performed assuming a glass fiber volume fraction of approximately 60%, using values from the literature for the elastic modulus of glass fibers and the polymer matrix. For the rule-of-mixtures prediction, an orientation/efficiency factor (η=0.25) was introduced to account for the short and randomly oriented nature of the recycled fibers. Fracture tests were performed by means of a CEAST Resil Impactor Charpy pendulum (ITW group, Glenview, IL, USA) equipped with a DAS 4000 Acquisition System, using an impact energy of 3.6 J, an impact speed of 1 m/s, and a span length of 48 mm. Samples with a notch depth-to-width ratio of 0.3 were fractured at room temperature. The selected notch geometry differs from the ISO 179 standard [32] recommendation (~0.5) and was used consistently for all specimens to ensure comparable fracture behavior in heterogeneous recycled composites. For each composition, five specimens were fractured, and the average and standard deviation values of impact resilience were calculated. Statistical analysis was performed using one-way analysis of variance (ANOVA) followed by Tukey’s HSD post hoc test to evaluate the significance of differences among the investigated formulations. Differences were considered statistically significant for *p* < 0.05.

## 3. Results and Discussions

### 3.1. GFRP Milling and Characterization

**Figure 1:**
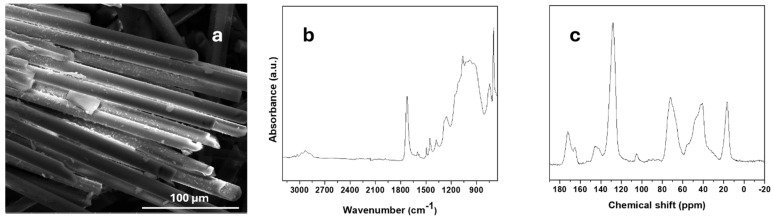
SEM image (**a**), FTIR analysis (**b**), and NMR spectrum (**c**) of calcinated glass fibers.

Fiber content determination in GFRP was performed through a material calcination procedure exploiting the degradation of the organic phase. The fiber-to-resin ratio was estimated to be 71 ± 1 wt%. Then, the chemical composition of the fibers was defined through EDX spectroscopy, as shown in Table 2 (the corresponding SEM image is shown in Figure 1a).

The detected elements and their relative amounts are consistent with the typical elemental composition of E-glass, the most common type used for fiber-based production, specifically the boron-free variant. The organic fraction of the GFRP was characterized using vibrational and NMR spectroscopic techniques. The absorption bands observed in the FTIR spectrum suggest that the material is an unsaturated styrene–polyester resin (Figure 1b). Specifically, the spectrum displays signals corresponding to hydroxyl stretching (a broad band between 3100 and 3500 cm^−1^), aromatic (3000–3100 cm^−1^) and aliphatic (2800–3000 cm^−1^) CH groups, as well as the absorbance of carbonyl groups at 1730 cm^−1^. Additional features include bands at ~1500 cm^−1^, supporting the presence of aromatic rings, and at ~1250 cm^−1^, attributable to ester groups. The intense band centered at approximately 1000 cm^−1^ originates from the absorption of the predominantly siliceous network of the glass fibers present in the analyzed sample.

The NMR analysis (Figure 1c) further contributed to the identification of the chemical groups constituting the resin monomers. The most intense resonance at ~128 ppm, together with a weaker signal at ~144 ppm, is attributable to aromatic ^13^C nuclei of styrene units and aromatic diacid moieties, most likely phthalic anhydride. These resonances are characteristic of protonated and quaternary aromatic carbons in substituted phenyl rings. The double peak observed in the 165–175 ppm region is assigned to distinct carbonyl carbons (C=O) of ester and anhydride functionalities, arising from phthalic and maleic anhydride units incorporated during the synthesis of the polyester precursor. The resonance at ~70 ppm is assigned to ester C–O carbons, with a possible contribution from secondary alcohol (C–OH) carbons. The broad and overlapping resonances in the 35–55 ppm region are associated with aliphatic backbone carbons formed during the styrene polymerization and the crosslinking of other unsaturated groups during curing, reflecting the structural heterogeneity of the network. Finally, the resonance at approximately 17 ppm is attributed to methyl carbons, most likely arising from propylene glycol units present in the polyester precursor formulation.

For the mechanical recycling of GFRP as a reinforcement in thermoplastic composites, fine grinding of the GFRP is required. The grinding and subsequent sieving stages are critical, since, as is well-known, the particle size distribution strongly affects both the processability of GFRP/polystyrene mixtures and the performance of the final materials [12,16]. In particular, excessively coarse inclusions can lead to local stress concentrations, compromising the mechanical strength of the composites, while excessive size reduction will inevitably diminish the reinforcing effect of the glass fibers, resulting in composites with limited mechanical properties.

**Figure 2:**
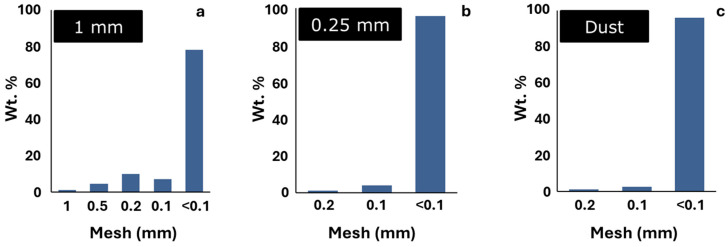
Particle size distribution in the ground materials obtained using 1 mm (**a**) and 0.25 mm (**b**) bottom screen during milling, and in the dust fraction (**c**).

The particle size distributions obtained by sieving are shown in Figure 2.

The sieving results indicate that, for all ground materials, a substantial fraction passed through the finest (0.1 mm) sieve, accounting for approximately 80% of the material ground with the 1 mm screen and about 95% for the materials ground using the finer screens.

It is worth noting that the labels “1 mm” and “0.25 mm” refer to the nominal bottom sieve aperture used during milling rather than to the actual fiber length. Due to the anisotropic morphology of the milled GFRP, fibers longer than the nominal mesh size may still pass through the sieve if their transverse dimension is sufficiently small and favorable orientation occurs during vibratory sieving.

**Figure 3:**
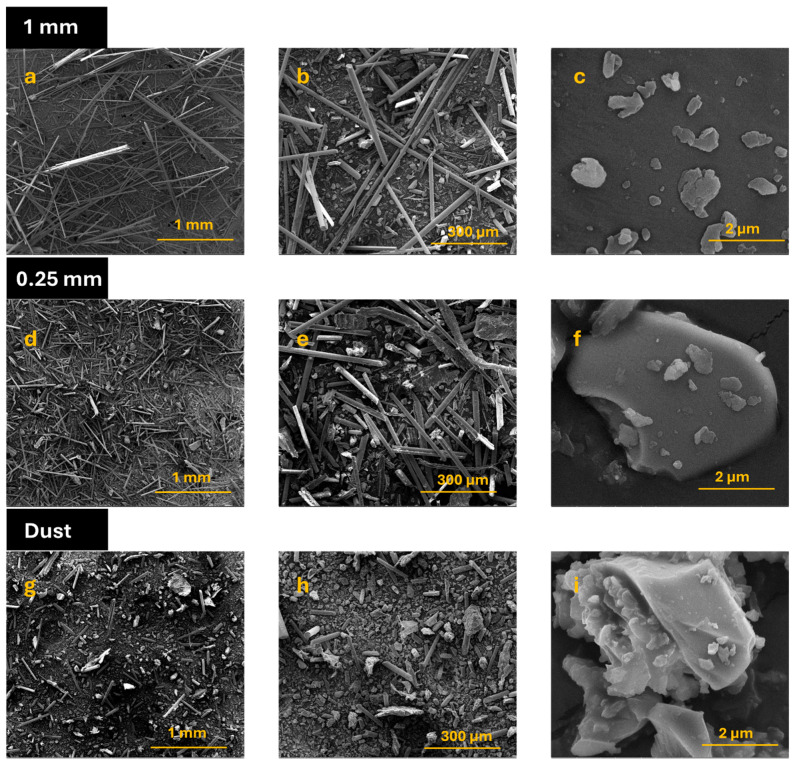
SEM micrographs of the milled materials: 1 mm (**a**–**c**), 0.25 mm (**d**–**f**), and cut powder (**g**–**i**). Images (**a**,**d**,**g**) were acquired at a scale bar of 1 mm; images (**b**,**e**,**h**) at 300 µm; and images (**c**,**f**,**i**) at 2 µm.

SEM provided detailed insight into the morphology of the three classes of milled GFRP materials (Figure 3), allowing us to evaluate the effects of milling and sieving stages on fiber preservation and particle fragmentation. The results clearly highlight a progressive transition from a fiber-dominated morphology to a predominantly particulate one, which lets us suppose a significant impact on the properties of the recycled materials.

In particular, the material ground with the 1 mm screen retained a morphology largely characterized by individual glass fibers and few residual fiber bundles, still embedded in the thermoset matrix, with high aspect ratios, many of which exhibited lengths exceeding 1 mm (Figure 3a–c). This morphology suggests that the milling conditions were sufficiently mild to preserve a substantial fraction of the original fiber length, which is a key requirement for effective stress transfer in composites. At the same time, the presence of coarse and isodimensional particles, mainly associated with thermoset matrix fragments and surface coating residues, indicates incomplete comminution of the non-fibrous phase. However, these particles can be efficiently removed by sieving, mitigating their potential role as stress concentrators in the final composite [33].

Concerning milling using the 0.25 mm screen, a noticeable reduction in fiber length to values generally below 500 µm can be evidenced (Figure 3d–f). Despite this size reduction, fibers and small bundles still retained a high aspect ratio and were able to pass through the 100 µm sieve mesh. This morphology represents a compromise condition, in which fiber fragmentation is more pronounced but the material still maintains a significant fibrous character, potentially balancing reinforcement efficiency and processability. In contrast, the cutting powder exhibited a morphology dominated by micrometric, isodimensional particles, with only a limited fraction of highly fragmented fibers with lengths of about a few hundred micrometers (Figure 3g–i). This extensive fiber breakage severely reduced the effective aspect ratio, thereby limiting the reinforcing contribution of the glass fibers and shifting their role from reinforcement toward that of a conventional mineral filler.

On these bases, sieving prior to material production was deemed necessary to remove the coarser fractions, where present, reducing the heterogeneity of the milled materials. Therefore, the following fractions (yield > 95 wt%) were considered for the subsequent composite production:

- Milled 1 mm, fraction < 500 µm (label “1 mm”).
- Cutting powder, fraction < 100 µm (label “Dust”).

For the 0.25 mm fraction (label “0.25 mm”), no separation through sieving was adopted.

The estimated aspect ratio distributions reported in Table 3 highlight a clear morphological transition across the granulometric fractions. The 1 mm fraction is characterized by highly elongated fibers with a mean aspect ratio of approximately 50, while the 0.25 mm fraction exhibits intermediate values around 22, reflecting a mixed population of fibers and shorter fragments. In contrast, the dust fraction shows near-equiaxed particles with aspect ratios slightly higher than 1. The median and weighted aspect ratio are also reported to better represent the typical particle morphology and to further emphasize the role of the longest fibers, which dominate the load-bearing response, when embedded into a polymer matrix.

### 3.2. Composite Characterization

The composites were prepared as described in the experimental section, and 50 wt% and 75 wt% of either one of the ground GFRP fractions or dust were added to polystyrene.

The thermal behavior of PS and of the composites containing 75 wt% of the different GFRP fractions was evaluated by DSC in order to assess possible effects of the high GFRP loading on the polymer glass transition temperature (T_g_, Table 4). All composite samples showed a Tg very close to the value observed for PS. The negligible variation in T_g_ indicates that the incorporation of a high amount of GFRP filler did not significantly affect the segmental mobility of the polystyrene matrix. This result suggests that, despite the extremely high filler content, no appreciable modification of the thermal response of the polymer phase occurred within the investigated system. In particular, the solvent-assisted cold-mixing process appeared to preserve the intrinsic thermal behavior of the recycled polymer matrix, with no evidence of plasticization or thermal destabilization induced by the presence of the recycled GFRP fraction.

In Table 5, the mechanical parameters of composites, calculated from flexural and impact tests, are presented. As can be observed, the incorporation of GFRP leads to a significant modification of the recycled PS mechanical parameters. In fact, composites exhibit higher elastic modulus (up to 9800 MPa for PS_75_0.25mm) and peak stress values (up to 58 MPa for the same sample) with respect to unfilled PS (1400 MPa and 19 MPa, respectively). Conversely, neat PS displays a substantially higher strain at break (about ten times higher than all composites) and greater impact resilience (approximately twice that of PS_75_1mm).

These results demonstrate that both filler size and content strongly influence the mechanical performance of the composites. For both the PS_50 and PS_75 systems, the use of 0.25 mm particles results in higher elastic modulus and peak stress compared to 1 mm fillers (e.g., PS_50_0.25 mm: 6700 ± 200 MPa and 57 ± 4 MPa vs. PS_50_1 mm: 6100 ± 200 MPa and 50 ± 6 MPa). This behavior can be attributed to the higher specific surface area of finer particles, which promotes more efficient stress transfer at the interface and reduces the presence of defects typically associated with coarser fractions. Although the 1 mm fibers possess a higher aspect ratio, which would theoretically enhance reinforcement efficiency, composite performance is also strongly dependent on fiber dispersion. Under the adopted processing conditions, the relatively long fibers are more prone to entanglement and clustering during mixing. Furthermore, the lower viscosity of the system may limit the shear stresses required for effective fiber separation and distribution. As a result, the potential reinforcement benefit associated with the higher aspect ratio is partially offset by poorer dispersion, leading to the lower mechanical properties observed experimentally. Increasing the filler content from 50 wt% to 75 wt% leads to a significant increase in stiffness, with the modulus rising from approximately 6100–6700 MPa to 8600–9800 MPa. Peak stress follows a similar trend, while the strain at break decreases from about 0.9% to 0.6–0.7%, consistent with the typical behavior of highly filled polymer systems, where increased stiffness is accompanied by reduced ductility and a more brittle mechanical response.

Moreover, for the best-performing composite in terms of measured modulus, i.e., PS_75_0.25mm, benchmarking against micromechanical models was performed. The upper-bound rule-of-mixtures prediction is approximately 43 GPa, while the effective modulus considering fiber orientation is about 10–11 GPa. The Halpin–Tsai model yields values in the range of 8–12 GPa, in good agreement with the experimental modulus of 9800 MPa.

Composites produced with dust fractions show the lowest modulus and peak stress (e.g., PS_50_dust: 5100 ± 100 MPa and 43 ± 4 MPa), suggesting that excessively fine particles may promote void formation or weak interfacial regions, thereby compromising structural integrity. A comparable trend is observed in impact resilience, which increases with filler loading and decreases with particle size, following the sequence dust < 0.25 mm < 1 mm; however, all systems retain the intrinsically brittle character of the PS. Although longer fibers (0.25 mm and 1 mm) can partially hinder crack propagation and provide a slight improvement in resilience, the overall response remains limited, with dust-based samples showing particularly low values (0.8 ± 0.1 kJ/m^2^).

One-way ANOVA confirmed the experimental trends, with significant differences among formulations observed for all investigated mechanical properties, as further supported by Tukey’s HSD post hoc test. From a mechanical standpoint, the reduction in aspect ratio directly affects the reinforcing efficiency (see Table 5). High-aspect-ratio fibers are more effective in stress transfer and crack bridging, contributing to increased stiffness and strength. Conversely, low-aspect-ratio particles primarily act as fillers, with limited ability to carry load.

Based on these findings, the composite containing 0.25 mm particles at the highest filler content emerges as the most promising candidate for further investigation, particularly through interfacial modification strategies aimed at enhancing fiber–polystyrene adhesion and overall performance.

**Figure 4:**
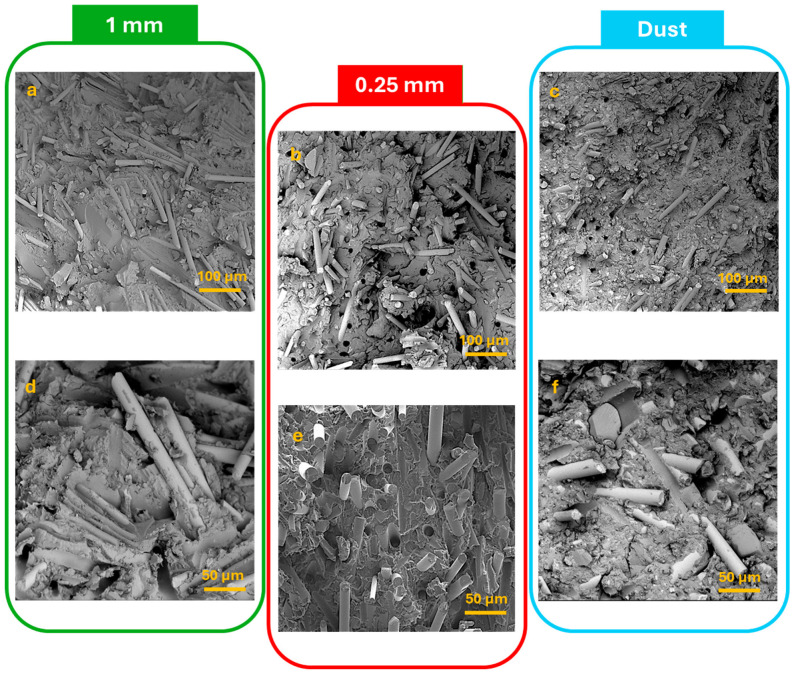
SEM low- and high- magnification micrographs of the fracture surfaces of PS-based composites containing 75% GFRP of 1 mm (**a**,**d**), 0.25 mm (**b**,**e**) and Dust (**c**,**f**).

Morphological analysis of the polystyrene-based composites was performed on the fractured surfaces of the samples tested under impact. In Figure 4a–c, low-magnification micrographs are shown to illustrate the general appearance of the surfaces of composites with a high GFRP content, while Figure 4d–f show the corresponding high-magnification images.

Morphological analysis highlights a strong dependence of dispersion and interfacial features on filler size. Composites with 1 mm GFRP (Figure 4a,d) show a heterogeneous fiber distribution, with fibers poorly embedded in the PS, indicative of weak interfacial adhesion and limited stress transfer efficiency. Fractographic evidence suggests preferential crack propagation along the fiber–PS interface, consistent with interfacial debonding and contributing to the modest increase in impact resilience. Reducing the filler size to 0.25 mm (Figure 4b,e) results in a more homogeneous distribution of the fibers and a lower occurrence of debonding, although interfacial voids still indicate poor adhesion. Dust-based systems (Figure 4c,f) exhibit the highest degree of dispersion, with a dense population of micrometric particles uniformly distributed within the PS.

These microstructural features rationalize the mechanical response, which, at the high filler load used, is the result of a balancing among the aspect ratio of the fibrous fraction, surface area of the ground GFRP, dispersion, and interfacial adhesion. The 1 mm fraction, whose long fibers are organized in bundles or interlocked, results in composites with a lower mechanical performance with respect to the 0.25 mm fraction, where fibers are shorter but have better dispersion. The dust fraction, on the other hand, is reasonably well dispersed, but the very high surface with low adhesion to the matrix reduces the overall load-bearing ability of the composite.

The interfacial interaction between GFRP particles and the polystyrene phase is expected to be governed primarily by mechanical interlocking and frictional stress transfer, as no chemical compatibilization was introduced. The adhesion between glass fibers and the polymeric matrix is, therefore, limited, and load transfer efficiency depends strongly on fiber surface condition, particle morphology, and dispersion state. Under applied stress, interfacial debonding and fiber pull-out are likely to occur, as also suggested by the observed fracture features [34]. These mechanisms contribute to energy dissipation but simultaneously limit the maximum achievable stress transfer, thus explaining the differences in mechanical performance among the investigated systems. In addition, the heterogeneous nature of the recycled GFRP particles may further enhance stress concentration zones, locally weakening the interface and promoting premature failure.

Finally, the processability of these highly filled composites using conventional polymer processing technologies was investigated as a potential additional recycling step for previously recycled materials [35]. For this purpose, the material with the highest fiber content (0.25 mm in size) was selected and melt-processed as described in the experimental section. This choice is based on a twofold consideration: on the one hand, this material exhibits the most promising properties; on the other hand, its high fiber content represents the most critical condition for melt processing.

**Figure 5:**
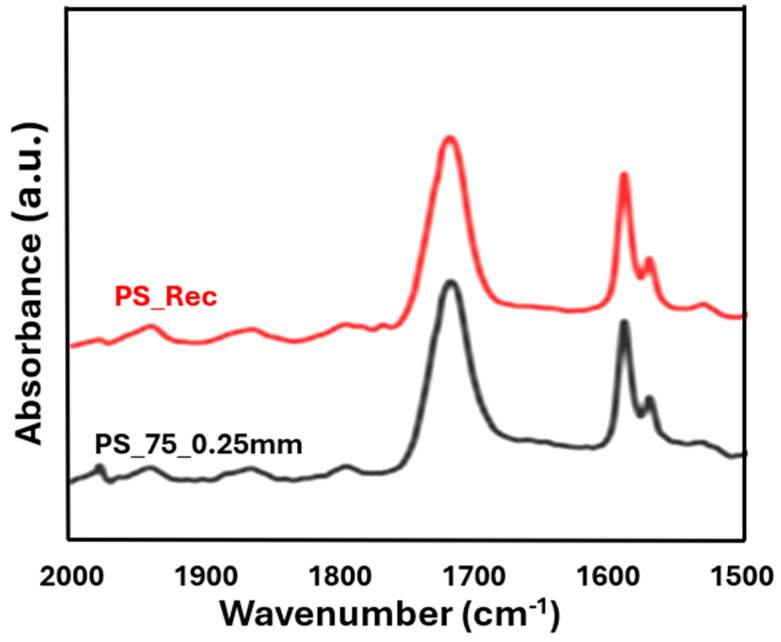
FTIR spectra of the polystyrene-based materials before (black curve) and after (red curve) recycling.

In Figure 5, the FTIR spectra of the composites before and after melt mixing are shown, respectively.

The comparison between the spectra shows no appreciable changes in the carbonyl region after processing, suggesting the absence of significant thermal degradation phenomena. In particular, no variations in intensity or the appearance of new signals are observed in the carbonyl region (1650–1750 cm^−1^), which corresponds to the chemical groups commonly associated with the thermo-oxidative degradation of many polymers.

The mechanical parameters of the recycled materials obtained from flexural and impact tests are reported in Table 6, together with those of the pre-recycled materials for comparison.

Recycling induces a measurable but limited reduction in mechanical performance. The elastic modulus decreases from approximately 9800 MPa to 9600 MPa, while peak stress drops from 58 MPa to 50 MPa; impact resilience is more affected, reaching about 1.4 kJ/m^2^. This moderate degradation can be rationalized by considering the initial morphology of the reinforcement: the GFRP phase is already characterized by medium-to-short, highly fragmented fibers, so the additional shear stresses imposed during melt reprocessing are unlikely to cause significant further fiber shortening. As a result, the reinforcing efficiency is largely preserved, and the recycled composites retain most of their load-bearing capability. Moreover, the absence of detectable thermo-oxidative degradation indicates that the polymer phase maintains its chemical integrity throughout processing. Overall, polystyrene-based composites subjected to recycling exhibit only minor losses in stiffness and strength while preserving their structural and functional characteristics, confirming their suitability for conventional reprocessing routes and highlighting their potential for sustainable reuse.

## 4. Conclusions

Highly loaded GFRP-filled polystyrene-based composites were successfully prepared by a solvent-assisted cold-mixing process and subsequently characterized from chemical, morphological, and mechanical perspectives. First, the grinding process of GFRP represents a key step in defining the properties of the recycled materials. Particle size distribution is indeed the variable that most strongly influences the composites’ properties, both due to the effect of glass fiber length on mechanical performance (longer fibers lead to higher-performing composites) and the issues that the finer fraction can cause during mixing and in terms of impact resistance. The 1 mm ground fraction remains processable after sieving with 0.5 mm meshes, which is necessary to remove the coarser fragments. The solvent-assisted mixing process proves particularly advantageous for very fine fractions, such as the powder generated during grinding and cutting, or when incorporating a very high amount of GFRP into the polystyrene. Once effective mixing has been achieved through the solvent-assisted process, the composite material can be melt-processed while maintaining an adequate dispersion of GFRP, with no tendency toward re-aggregation. The recycling of polystyrene-based composites containing GFRP is, therefore, feasible, albeit with some reduction in mechanical performance that can be attributed to the inevitable fragmentation of the glass fibers, as no alteration in the spectroscopic profile was observed, consistent with no significant chemical modification of the polymer component. Recycled polystyrene-based composites were found to preserve most of their initial mechanical and chemical characteristics, and no signs of thermo-oxidative damage were detected. Although recycling leads to some reduction in stiffness, strength, and impact performance, these decreases are relatively modest, and the materials can still be effectively processed using standard industrial melt-processing techniques. This demonstrates that the composites retain a substantial portion of their original mechanical properties, highlighting a promising route for their sustainable reutilization.

## Figures and Tables

**Table 1:** Cold-mixed composites containing GFRP.

Code	Polystyrene Content (wt%)	GFRP Content (wt%)
**PS**	100	0
**PS_50_1mm**	50	50
**PS_50_0.25mm**	50	50
**PS_50_dust**	50	50
**PS_75_1mm**	25	75
**PS_75_0.25mm**	25	75
**PS_75_dust**	25	75

**Table 2:** EDX elemental analysis of GFRP calcinated fibers.

Elemental Composition (wt%)								
O	Na	Mg	Al	Si	Ca	Ti	Fe	Cu
55.0 ± 3.1	0.6 ± 0.1	1.2 ± 0.3	5.8 ± 0.7	21.4 ± 1.8	15.1 ± 1.1	0.3 ± 0.1	0.3 ± 0.1	0.2 ± 0.1

**Table 3:** Estimated L/D distributions for different granulometric fractions based on SEM observations.

Fraction	Mean Aspect Ratio	Median Aspect Ratio	Weighted Aspect Ratio
**1 mm**	50 ± 15	42 ± 12	60 ± 20
**0.25 mm**	22 ± 8	15 ± 6	28 ± 10
**Dust**	2.2 ± 0.8	1.6 ± 0.5	2.5 ± 1.0

**Table 4:** T_g_ of PS and PS_75_0.25mm, as determined by DSC from the second heating scan.

Code	T_g_ (°C)
**PS**	102.7
**PS_75_1mm**	103.3
**PS_75_0.25mm**	103.6
**PS_75_dust**	103.2

**Table 5:** Flexural and impact mechanical parameters of polystyrene-based composites.

Code	Modulus (MPa)	Peak Stress (MPa)	Strain at Break (%)	Impact Resilience (kJ/m^2^)
**PS**	1400 ± 300	19 ± 3	10.1 ± 0.9	5.2 ± 0.5
**PS_50_1mm**	6100 ± 200	50 ± 6	0.9 ± 0.1	1.7 ± 0.1
**PS_50_0.25mm**	6700 ± 200	57 ± 4	0.9 ± 0.1	1.4 ± 0.1
**PS_50_dust**	5100 ± 100	43 ± 4	0.9 ± 0.1	0.8 ± 0.1
**PS_75_1mm**	8700 ± 200	51 ± 6	0.7 ± 0.1	2.7 ± 0.2
**PS_75_0.25mm**	9800 ± 500	58 ± 3	0.6 ± 0.1	2.0 ± 0.1
**PS_75_dust**	8600 ± 200	49 ± 8	0.6 ± 0.1	0.8 ± 0.1

**Table 6:** Flexural and impact mechanical parameters of the polystyrene-based composites, both before and after recycling. All values are expressed as mean ± standard deviation based on five independent specimens (n = 5).

Code	Modulus (MPa)	Peak Stress (MPa)	Strain at Break (%)	Impact Resilience (kJ/m^2^)
**PS_75_0.25mm**	9800 ± 500	58 ± 3	0.6 ± 0.1	2.0 ± 0.1
**PS_Rec**	9600 ± 200	50 ± 5	0.5 ± 0.1	1.4 ± 0.2

## Data Availability

The data presented in this study are available from the corresponding author upon reasonable request.
